# Developing eThread Pipeline Using SAGA-Pilot Abstraction for Large-Scale Structural Bioinformatics

**DOI:** 10.1155/2014/348725

**Published:** 2014-06-09

**Authors:** Anjani Ragothaman, Sairam Chowdary Boddu, Nayong Kim, Wei Feinstein, Michal Brylinski, Shantenu Jha, Joohyun Kim

**Affiliations:** ^1^RADICAL, ECE, Rutgers University, New Brunswick, NJ 08901, USA; ^2^Center for Computation and Technology, Louisiana State University, Baton Rouge, LA 70803, USA; ^3^Department of Biological Sciences, Louisiana State University, Baton Rouge, LA 70803, USA

## Abstract

While most of computational annotation approaches are sequence-based, threading methods are becoming increasingly attractive because of predicted structural information that could uncover the underlying function. However, threading tools are generally compute-intensive and the number of protein sequences from even small genomes such as prokaryotes is large typically containing many thousands, prohibiting their application as a genome-wide structural systems biology tool. To leverage its utility, we have developed a pipeline for eThread—a meta-threading protein structure modeling tool, that can use computational resources efficiently and effectively. We employ a pilot-based approach that supports seamless data and task-level parallelism and manages large variation in workload and computational requirements. Our scalable pipeline is deployed on Amazon EC2 and can efficiently select resources based upon task requirements. We present runtime analysis to characterize computational complexity of eThread and EC2 infrastructure. Based on results, we suggest a pathway to an optimized solution with respect to metrics such as time-to-solution or cost-to-solution. Our eThread pipeline can scale to support a large number of sequences and is expected to be a viable solution for genome-scale structural bioinformatics and structure-based annotation, particularly, amenable for small genomes such as prokaryotes. The developed pipeline is easily extensible to other types of distributed cyberinfrastructure.

## 1. Introduction

Modern systems biology holds a significant promise to accelerate the development of personalized drugs, namely, tailor-made pharmaceuticals adapted to each person's own genetic makeup. Consequently, it helps transform symptom-based disease diagnosis and treatment to “personalized medicine,” in which effective therapies are selected and optimized for individual patients [1]. This process is facilitated by various experimental high-throughput technologies such as genome sequencing, gene expression profiling, ChIP-chip/ChIP-seq assays, protein-protein interaction screens, and mass spectrometry [2–4]. Complemented by computational and data analytics techniques, these methods allow for the comprehensive investigation of genomes, transcriptomes, proteomes, and metabolomes, with an ultimate goal to perform a global profiling of health and disease in unprecedented detail [5].

High-throughput DNA sequencing, such as Next-Generation Sequencing (NGS) [6–8], is undoubtedly one of the most widely used techniques in systems biology. By providing genome-wide details on gene sequence, organization, variation, and regulation, NGS provides means to fully comprehend the repertoire of biological processes in a living cell. Importantly, continuing advances in genome sequencing technologies result in rapidly decreasing costs of experiments making them affordable for individual researchers as well as small research groups [8]. Nevertheless, the substantial volume of biological data adds computational complexity to downstream analyses including functional annotation of gene sequences of a donor genome [9]. Consequently, bioinformatics components of systems biology pipelines are subject of intense research oriented on improving their accuracy in interpreting and analyzing raw NGS data, as well as on the development of effective computing strategies for processing large amounts of data.

One of the major challenges in NGS analytics is a reliable proteome-wide function inference of gene products. This is traditionally accomplished using sequence-based methods, which annotate target proteins by transferring molecular function directly from homologous sequences [10, 11]. Despite a high accuracy of these methods within the “safe zone” of sequence similarity, their applicability to the “twilight zone” is more complicated due to ambiguous and equivocal relationships among protein sequence, structure, and function [12]. It has been shown that relaxing sequence similarity thresholds in function inference inevitably leads to high levels of misannotation [13]. Therefore, low false positive rates can be maintained only at the expense of a significantly reduced coverage, which, in turn, hinders the development of systems-level applications. To address this issue, combined evolution/structure-based approaches to protein functional annotation have been developed [14–16]. Integrating sequence and structural information yields an improved performance within the “twilight zone” of sequence similarity, which significantly extends the coverage of targeted gene products. Furthermore, these methods consider many aspects of protein molecular function including binding to small organic molecules, inorganic groups, for example, iron-sulfur clusters and metal ions, and interactions with nucleic acids and other proteins [17]. Structural bioinformatics approaches offer certain advantages over pure sequence-based methods; however, these algorithms also present significant challenges in the context of their practical implementation. Compared to ultra-fast sequence alignments and database searches using, for example, BLAST [18], protein threading and metathreading that include structure-based components put significantly higher demands for computing resources, which becomes an issue particularly in large, proteome-scale projects.

The last decade has seen a growing interest in using distributed cyberinfrastructure (DCI) for various bioinformatics applications [19–21]. For example, the MapReduce programming model along with Hadoop, introduced initially for massive distributed data processing, was explored [21–23]. Also, cloud environments are increasingly becoming popular as a solution for massive data management, processing, and analysis [19, 20, 24]. Previously, SAGA-Pilot-based MapReduce and data parallelization strategies were demonstrated for life science problems, in particular, such as alignment of NGS reads [20, 25, 26]. Despite the successful cloud-oriented implementations of various bioinformatics tools, significantly fewer studies focused on the porting of complex structural bioinformatics algorithms to distributed computing platforms.

In this work, we present a pilot-based implementation of metathreading for the structural and functional characterization of large datasets of gene products. Specifically, we developed a pipeline for eThread, a recently developed metathreading approach [27], tailored primarily for the Amazon EC2 distributed computing infrastructure and also easily extensible for other types of DCIs. eThread integrates ten state-of-the-art single threading algorithms to accurately identify template proteins, which can be subsequently used in both structure modeling and functional annotation. The latter covers a broad spectrum of protein molecular function, including ligand, metal, inorganic cluster, protein, and nucleic acid binding [17]. Since eThread features a diverse collection of algorithms, its deployment on large multicore systems necessarily requires comprehensive profiling to design an efficient execution strategy. In our previous study, we performed a rigorous profiling of eThread in terms of time-to-solution and memory footprint, focusing on the optimal utilization of resources provided by homogeneous high-performance computing (HPC) clusters [28]. In contrast to HPC machines, which are typically composed of a large number of identical nodes, modern cloud computing infrastructures, such as Amazon EC2, provide a wide selection of instance types comprising varying combinations of CPU, memory, storage, and networking capacity. These on-demand instances have different hourly rates; therefore, in addition to time-to-solution, the efficient processing of large biological datasets using commercial cloud computing platforms should take account of the overall cost-to-solution as well. In this study, we report an effective implementation of metathreading using a pilot-based multilevel scheduling. This approach offers significant advantages in terms of the data, job, and failure management in large-scale structural bioinformatics applications.

This paper is organized as follows. Introductions of eThread, Amazon EC2, and SAGA-based pilot framework are presented. Then, our pipeline is described along with key strategies for parallelization. Benchmark results revealing characteristics of computational tasks required for the eThread pipeline as well as those associated with EC2 infrastructure are presented. We, then, discuss our contributions and future directions, which are followed by concluding remarks.

## 2. Materials and Methods

**Figure 1:**
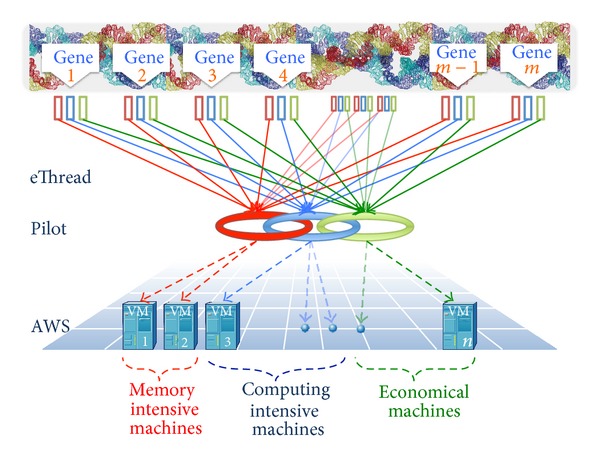
Schematics of the pilot-based eThread pipeline on EC2. The eThread pipeline can accept a massive number of sequences, identified from genome-wide sequencing methods such as RNA-Seq, for example, as input, and carry out metathreading-based structural bioinformatics analysis including structure modeling. SAGA-Pilot enables its execution on Amazon EC2 cloud environment to be efficient by facilitating data and task-level parallelization.

As schematically shown in Figure 1, the eThread pipeline on EC2 aims to carry out genome-scale structural bioinformatics analysis efficiently using the SAGA-based pilot framework. Here, we describe our methods and backgrounds for the developed pipeline and benchmark experiments.

### 2.1. eThread, a Scientific Application Comprising Multiple Standalone Tools

eThread is a metathreading tool and was developed for predicting protein structure and function, whose input is a protein sequence [17, 27]. Unlike other tools based on sequence-based approaches, eThread is template-based. Template structures are identified in the PDB library using metathreading that combines the 10 individual threading/fold recognition algorithms. The machine-learning-based meta-analysis is carried out using all outputs from these 10 threading programs. The overall pipeline of eThread is, therefore, a two-step pipeline. The first step runs the 10 state-of-the-art threading/fold recognition algorithms, CS/CSI-BLAST, COMPASS, HHpred, HMMER, pfTools, pGenTHREADER, SAM-T2K, SP3, SPARKS, and THREADER. NCBI BLAST is also needed for construction of sequence profiles for most of tools and PSIPRED is required for the prediction of secondary structure that is a preprocessing task for THREADER. We summarize the ten tools in Table 1. While three-dimensional structures can be constructed by either MODELLER or TASSER-Lite using the output from eThread metathreading analysis, in this work, we only confine our focus on the metathreading analysis. The effective combination of multiple algorithms considerably extends the coverage of target sequences by distantly related templates and increases the overall modeling accuracy. According to the previous work [27], eThread systematically detects more structure templates than any single algorithm producing reliable structural models for 60–70% of all gene products across a given proteome.

Due to nature of the key strategy, that is, metathreading, eThread is a complicated tool requiring the execution of the 10 different threading tools that also could contain other tools and have dependency for the template library. Note that these tools were developed by different developers and their implementation strategies are all different and heterogeneous, which challenges an efficient execution of eThread. Previously, profiling of the individual tools using a single system was reported [28], and here we briefly summarize the results. The computational loads and the memory requirement for each threading tool were examined using a set of 110 sequences whose length is distributed between 50 and 600 amino acids. According to the results, we categorize each tool as “High,” “Medium,” and “Low” for each category as summarized in Table 1. For the computational load, a tool is assigned as “Low” if the running time for 110 sequences is less than 1-2 hours, “Medium” is for tools taking about 10 hours, and “High” is for tools taking more than 10 hours. Notably, THREADER requires about 5–19 hours for the data set, which makes the tool stand out in terms of the running time denoted as “Highest.” For the memory requirement, “High” is for tools needing more than 3 GB, “Medium” requires between 0.6 and 3 GB, and “Low” is tools requiring up to 0.6 GB. Interestingly, the memory requirement is highly dependent upon the use of BLAST in each tool. The reason why THREADER does not need a lot of memory is that we use our modified version that separates BLAST tasks out of the tool.

The basic structure of eThread is further illustrated with the simplest serial algorithm in Algorithm 1. First of all, all threading tools have a similar internal workflow, comprising preprocessing, two main tasks performed against chain and domain template libraries, and postprocessing tasks. The preprocessing step varies among the tools, and, again, some require running other tools such as BLAST or PSIPRED to prepare their input for the main tasks. Notably, all of threading tools are not developed to support any parallelism for multicore or multinode systems, implying that data parallelization would be a good strategy in the case of multiple sequences and that task-level parallelization that runs concurrently independent subtasks is desirable as indicated in the loops of Algorithm 1. BLAST has been available as multithreading or MPI-based, but for the simplicity and a practical reason (relatively low portion for the total time-to-solution), a single or two-thread execution is only considered. Regarding BLAST tasks, it is worth noting that tools such as THREADER, COMPASS, and SAMT2K invoke BLAST as a one-time preprocessing step, whereas HHpred, SP3/SPARKS, and pGenTHREADER are implemented to contain it within iterative tasks. This means that BLAST can run separately for the former three tools, whereas the latter three tools are difficult to separate BLAST. Taken together, in spite of common structures among the 10 threading tools, significant challenges exist for an optimal execution due to the difficulty of customizing possible task-level and data parallelization for each tool, which is further complicated by significant overhead stemming from the heterogeneous nature of EC2 infrastructure.

### 2.2. Amazon EC2 Infrastructure

Amazon provides the EC2 cloud computing environment which is an IaaS cloud [20]. This infrastructure is, in many ways, promising for large-scale scientific applications such as eThread, but distinctively different from traditional HPC environments. For example, Amazon Machine Image (AMI) is easily created, reusable, and maintained, consequently lowering the cost of installation and maintenance of required standalone tools. This is greatly beneficial for most of bioinformatics pipelines that are often composed of many open source tools whose development, update, and extension are nonuniform and frequent and have no connection to each other. For instance, threading tools are often developed with a specific OS environment, mostly Linux-like Oss, but developers could not test many different OS variants. We found that some tools such as SP3, SPARKS, and SAMT2K were not easily installed with Ubuntu but had no issue with CentOS or RedHat. Therefore, an easy way to avoid hassles associated with a compatibility issue is to create a specific AMI best for each threading tool with the most favorable Linux distro, which is likely to be the same one used by the developers, and then to reuse it for upcoming new releases of the tool.

For the eThread pipeline development, we chose to build one AMI configured for each single threading tool along with other additional programs needed, but it is also possible to install multiple tools in a single AMI. EC2 provides multiple types of instances and an end user is charged depending upon types of instances, running times, and storage options. Instance types are different in types of cores, the number of cores, memory sizes, instance storage, and network performance. The instances we used for this study are summarized in Table 2 and are chosen to represent several different categories such as economical, general, memory-optimized, compute-optimized, and storage-optimized cases. The external storage option is also a factor for the payment, and we need to use S3 and EBS as described in more detail later. It is also noted that while instances as a cluster are available from EC2, the developed pipeline, as powered by SAGA-Pilot, is able to execute large-scale calculations by coordinating individual VMs without a cluster environment. In summary, on-demand computing promised by IaaS cloud such as EC2 has a great potential for large-scale scientific applications and a lot of benefits once a user considers carefully the effective use of complex and heterogeneous infrastructure comprising many different instance types.

### 2.3. SAGA-Pilot Abstraction

An efficient utilization of distributed cyberinfrastructure is essential for a distributed application such as our eThread pipeline [20]. SAGA-Pilot abstraction provides an effective decoupling between the compute oriented tasks and associated data management [29, 30]. This alleviates the burden of the application to be confined with a particular resource for scheduling compute and data units. BigJob is a SAGA- (Simple API for Grid Applications-) based pilot framework which utilizes a Master-Worker coordination model [31]. It comprises high-level and easy-to-use APIs for accessing distributed resources and provisioning of job submission, monitoring, and more. It has been successfully utilized for efficient executions of loosely coupled and embarrassingly parallel applications on distributed cyberinfrastructure [25, 26]. BigJob has three major components. First, Pilot-Manager is responsible for the orchestration of pilots (Pilot-Compute and Pilot-Data) which run locally or on remote resources for assigned tasks. Pilot-Manager maps a data unit to a compute unit. BigJob is built upon SAGA Job API which invokes SAGA adaptors for submitting jobs to target resources while all details are hidden to BigJob level API. For this work, we use the Amazon Web Services adaptor, one of the many available SAGA adaptors. Second component is Pilot-Agent that collects local information of a system and is responsible for executing the compute unit(s) and placing the data units appropriately on the resource where the tasks are submitted. Finally, a coordination service, employing a redis server, helps in coordination and communication to facilitate the control flow and data exchange between Pilot-Manager and Pilot-Agent [32].

With Amazon EC2 infrastructure, a current SAGA-Pilot implementation handles the data management between tasks and S3 is configured to be used for the data store as default. In other words, any task once completed deposits predefined output into S3 storage and the next task is able to locate the output as its input.

Application workload management is also provided by Pilot APIs as follows. Pilot APIs comprise compute-unit and data-unit classes as primary abstraction. Using these, a distributed application can specify a computational task with input and output files [29, 32]. Once compute-units and data-units are submitted, they are queued at the redis-based coordination service and are processed recurrently by a scheduler. Importantly, Pilot-Manger's asynchronous interface allows an instantaneous response without delay, which facilities BigJob to complete the placement of compute/data-unit and thus is effective for dealing with a large number of tasks.

### 2.4. Benchmark Data Set

For all benchmarking experiments, manually curated 110 protein gene sequences whose lengths are distributed between 51 and 600 aa (amino acids) are prepared (see Table 3). These 110 sequences were used for runtime analysis of the EC2 instances against the 10 threading tools, the two additional tools, PSIPRED and BLAST, and meta-analysis similar to the previous work [28]. Most of benchmark experiments with the developed pipeline powered by SAGA-Pilot are carried out using 20 sequences chosen among 110 as described in Table 3.

## 3. Results

### 3.1. Development of Pilot-Based eThread Pipeline

**Figure 2:**
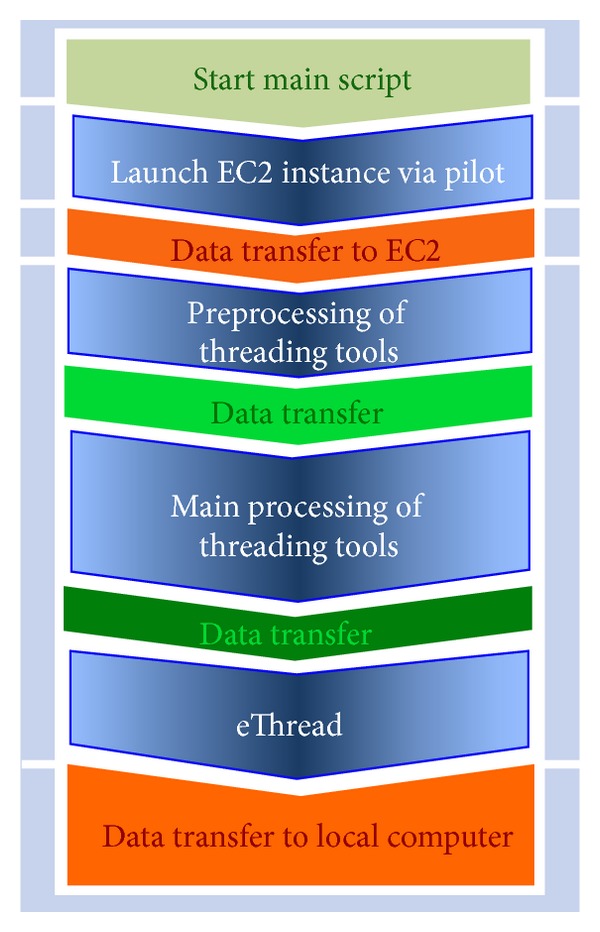
Overall workflow of the pilot-based eThread pipeline on EC2.

The schematic workflow of our developed pipeline is shown in Figure 2. The pipeline carries out four major steps that need to be taken sequentially for each input sequence. They are VM-launch, preprocessing, main processing, and eThread meta-analysis. Data transfer is needed between tasks and compute resources involved and is not examined in this work due to relatively insignificant contribution to the time-to-solution or the charge. For example, only less than 5 seconds are needed for moving 20 input sequences into EC2 and managed by the main script in the beginning of a pipeline job.

The main script of the pipeline, located in a local machine owned by a user, starts with an initialization of Pilot service instances, each of whom manages an EC2 VM creation, job submissions to the Pilot instance(s), and data transfer if needed. Importantly, SAGA-Pilot allows the pipeline to monitor the status of individual subtasks constituting eThread and thus can conduct the workflow efficiently to maximize the use of available resources on the fly while supporting various optimization scenarios. By exploiting this feature, data parallelization and task-level parallelization are easily implemented. For example, a simple task-level parallelism could be designed as shown in Algorithm 2. Multiple VMs are created and each VM or the number of VMs is assigned for tasks of each threading tool. By considering required workloads and computational requirements such as memory footprints, threading tools can be executed concurrently on proper VM(s). On the other hand, this simple parallelism scenario is likely to be inefficient if differences in threading tools and instances are significant. In this work, our primary contribution is to examine those multifaceted parameters associated with EC2, using 110 sequences as well as its subset, 20 sequences, and to demonstrate our pipeline capabilities toward the optimization solution of eThread execution.

### 3.2. Profiling EC2 Infrastructure Using 110 Sequence Benchmark

**Figure 3:**
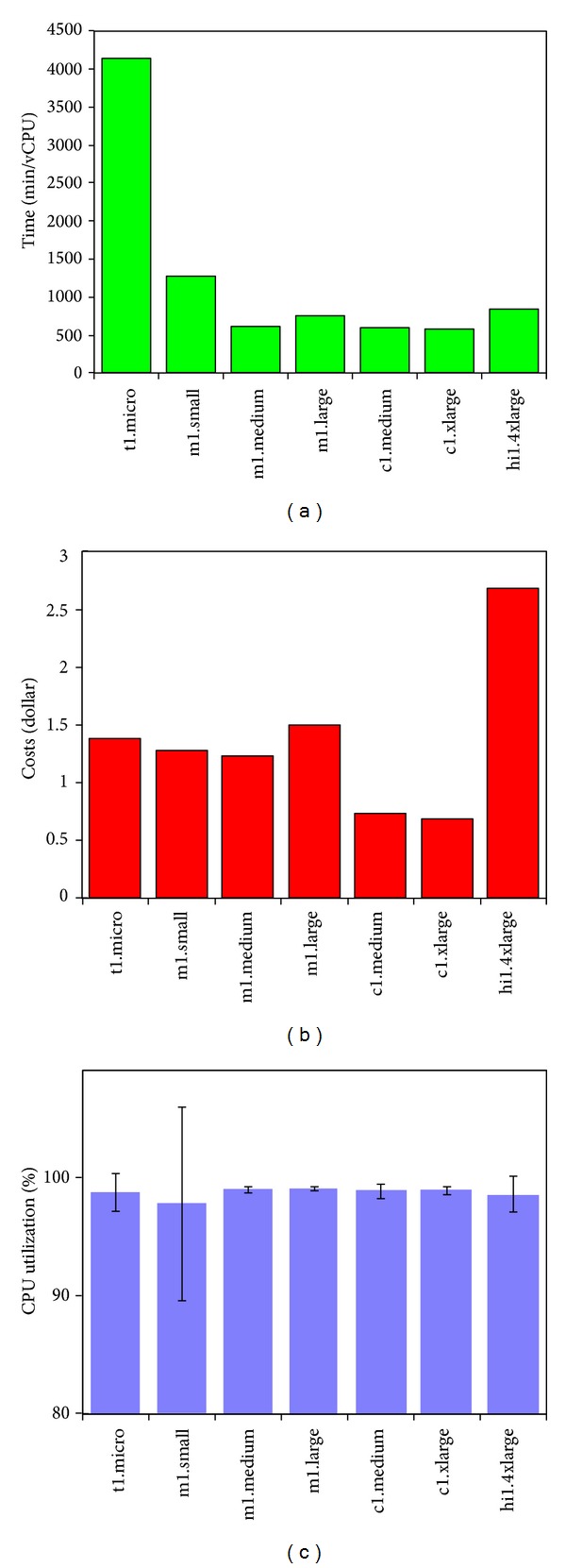
Required total execution times for 110 sequences shown in (a) and corresponding cost shown in (b) for pfTools across different types of EC2 instances. CPU utilization is also shown in (c).

How to run eThread on EC2 is critically important since the cost and the time-to-solution will increase considerably without optimization, and to some extent, making the pipeline unpractically expensive or time consuming for genome-scale analysis. Note that, due to the charging scheme from Amazon, two conditions for the optimization are not equivalent. For example, in Figure 3, the time-to-solution and the cost are compared when different instance types are used. The data shown is with pfTools and the 110 data set is used. We also report CPU utilization with error bars. The results are obtained by running command line scripts for each tool in a specific VM and thus reflect how a CPU core in each instance performs with respect to the time-to-solution and the cost. HMMER, CS/CSI-BLAST, THREADER, and pfTools are only tools requiring relatively small memory footprint and thus could run on all instances including t1.micro as shown. Our benchmark was completely carried out for 10 threading and two standalone tools, PSIPRED and BLAST. We found that the same trend is observed consistently among the results obtained for other tools (not shown). First of all, different cores in different instances are not the same; in particular, t1.micro is the slowest. While hi1.4xlarge is the most expensive one, obtained results indicate that a core in this instance seems to be slower than those in other instances such as the two c1 instances and even than m1.medium and m1.large. On the other hand, in terms of cost, hi1.4xlarge is worse, while t1.micro and two c1 instances ranked in the top list. Interestingly, the utilization of CPU is not always 100% as shown in the third figure of Figure 3, for which we will discuss more details later for possible explanations. t1.micro instance is somewhat different from other instances in many ways. It costs a lot less, and is often free with special promotion from Amazon, thus being adequate for running many subtasks, but the small memory, 0.6 GB, prohibits running many tools including SAM-T2K, pGenTHREADER, COMPASS, SPARKS, and SP3. Also, in spite of a possible execution for THREADER, the huge computing load, due to its underpowered capacity, prohibits practically its usage with this instance.

### 3.3. Profiling Computational Tasks for the eThread Pipeline on EC2

Contrast to the execution mode of eThread using a single computer system or a cluster system, the eThread pipeline implemented with SAGA-Pilot cannot avoid an overhead due to its underlying Master-Worker model. The overhead, first of all, arises from the data transfer between a local machine that runs the main script and remote compute resources in EC2 (indicated as orange in Figure 2) and the data exchange between elementary tasks managed by SAGA-Pilot, which is insignificant (data is not shown).

The coordination mechanism with SAGA-Pilot for tasks running in distributed resources is generally insignificant compared to main tasks associated with target applications of interest [20, 25, 30]. On the other hand, VM launch takes a certain amount of times and is unavoidable in our pilot-based implementations, which is, therefore, measured as a part of runtime analysis.

**Figure 4:**
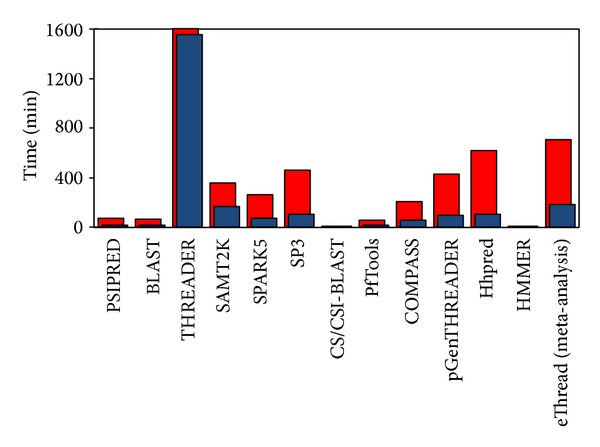
Pilot-based profiling of tools using different EC2 instances. Comparison of time-to-solution for the 10 threading tools, two standalone tools, BLAST and PSIPRED, and meta-analysis step is presented. Cases with m1.large (red) and hi1.4xlarge (blue) are shown and 20 sequences are used. Note that THREADER with m1.large takes 2897 mins which is not fully shown.

Profiling elementary subtasks in the workflow of the eThread pipeline is important for parallelization strategies. Using the pipeline, we conducted benchmark experiments to gain insights into relative computing loads across the tools against all instances we consider for this work. In Figure 4, we present the results comparing time-to-solutions across those tools when using m1.large and hi1.4xlarge. The input sequences are 20 sequences. In accordance with the previous work [28], the pipeline-based execution reveals a broad distribution of computational loads and memory requirements across the tools. Also, expected speed-ups, due to multicore utilization, are indicated. In particular, the execution of THREADER is, when hi1.4xlarge (16 cores) is used, now much reasonably down to about 1,660 min that becomes just two or three times more than time-to-solution of tools grouped as “High” in terms of computational loads. As reported in the previous work, the meta-analysis step of eThread does not need significant computing resource and is not expected to change much with different infrastructure. In fact, this step is not expected to be more optimized internally with task-level parallelism except data parallelization of input sequences. We will focus, primarily, on the optimization of the first step before this meta-analysis that comprises 10 threading tools and preprocessing steps.

Here, we would like to stress a possible future strategy, in order to gain more insights into the current underlying structure of eThread pipeline. As stated in the previous section, some tools such as THREADER, COMPASS, and SAMT2K need to run BLAST, but also further can be modified to run it in a separate subtask. In fact, THREADER, since the previous work [5], is already modified, resulting in, compared to COMPASS and SAMT2K, the fact that THREADER requires relatively lower memory footprint.

Additionally, the two main processing tasks of each treading tool against chain and domain libraries could be run separately, and this possible parallelization helps to achieve the overall optimization easily and significantly. We measure the portion of chain and domain tasks, and the results in the case of pfTools are presented in Table 4. Times for their postprocessing tasks as well as VM launch times are also reported. First of all, the relative portion between chain and domain is consistently found as 60% versus 40% across all instances, which is in accordance with the previous work [28]. However, t1.micro shows an exception such that the ratio is changed to 49% versus 51%. VM launch times are a bit fluctuating but its portion is insignificant except the cases with two expensive instances, c1.xlarge and hi1.4xlarge, since the speed-up for the main tasks is now decreased a lot due to multiple cores. In fact, the number of sequences, 20 in this benchmark, is far less than the number of sequences for a genome-scale analysis, and pfTools is a relatively less compute-intensive tool, implying the insignificant contribution from relative portions of VM launch as well as postprocessing tasks, compared to the main processing tasks.

### 3.4. VM Launch Time

While parallelization provides a chance of optimized execution, it is also true that the SAGA-Pilot-based pipeline running on EC2 cannot avoid some amounts of overhead associated with the use of distributed cyberinfrastructure and thus it is important to know how much they contribute. We carried out the dedicated experiments for measuring VM launch time and obtained results are presented in Table 5. In fact, VM launch time is affected by many factors and thus varies depending upon the conditions (e.g., compare the values reported in Table 4), but the range of fluctuations is typically a couple of minutes in general.

Overall, according to the experimental results, our benchmarks clearly show that the overhead arising from the use of SAGA-Pilot and the remote EC2 infrastructure is seemingly not significant, which is, in particular, becoming more justifiable as the size of computation with the pipeline is scaled up with more sequences and longer sequences.

### 3.5. eThread Pipeline and Its Optimal Execution

Presumably, the key question on how to implement an efficient execution of the eThread pipeline on EC2 infrastructure is directly related to the question on how to distribute subtasks on a heterogeneous resource pool.

**Figure 5:**
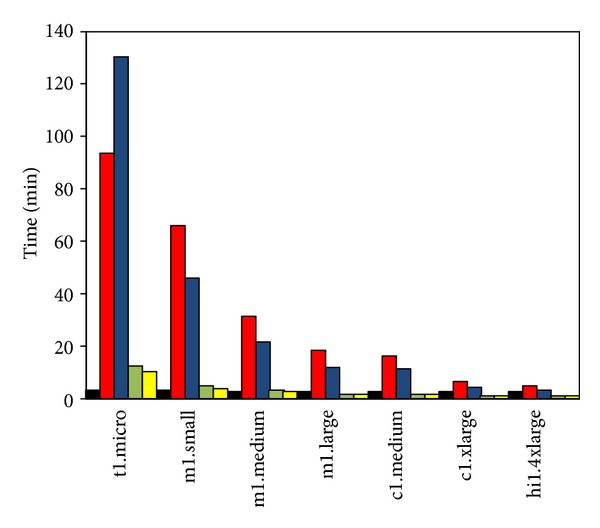
Time-to-solution of each elementary step in the pipeline using 2 VMs. Results are obtained with 20 sequences and pfTools are used. The times for VM launch (black), threading against chain library (red), threading against domain library (blue), postprocessing for chain (green), and postprocessing for domain (yellow) are shown together.

To demonstrate the agile capacity of our pipeline, we conducted more complex parallelization scenarios. First of all, two VMs are launched for each tool and results with 20 sequences are shown in Figure 5. As expected, more gains in time-to-solution are obtained since more cores from both VMs and the separate execution of the two main tasks are utilized to run multiple sequence calculations.

Apparently, it is not difficult to understand why the case of t1.micro-c1.xlarge outperforms other cases considering the inclusion of high performance 8-core c1.xlarge. On the other hand, the performance difference among other cases is not easy to predict, because the performance depends upon how subtasks are distributed and executed. When multiple sequences and multiple instances are considered, the key is to consider an efficient job distribution.

## 4. Discussion

### 4.1. eThread on EC2

Scientific applications such as eThread need large-scale computation as shown with 110 sequences. While often traditional HPC systems could be effective for such computational demands, many life science applications including eThread could find more benefits with cloud environments. Indeed, unlike scientific applications in other scientific domains, applications in life sciences are likely to be data-intensive and need to be implemented as pipelines, which makes HPC environments somewhat unfit. On-demand computing provided by EC2 is readily beneficial for data parallelization and task-level parallelization as examined with our pipeline for eThread in this work. Furthermore, the use of AMIs provides advantages for installation and maintenance of standalone tools and the AMIs are later reusable for other IaaS environment such as OpenStack-based clouds. SAGA-Pilot is an ideal solution to build such pipelines since it allows a rapid, powerful, and agile implementation for various and changing goals and strategies.

One of important challenges for the use of EC2 for eThread is to understand various factors of the IaaS infrastructure that affect the time-to-solution and the cost. For that purpose, we conducted benchmark experiments for estimating computational loads and corresponding costs and demonstrated the capability of our pipeline toward the optimization of its execution for massive input sequences.

First of all, in Table 6, the overall summary of benchmark results with respect to time-to-solution and cost-to-solution is presented. We conducted all possible combinations of threading tools and instance types shown in Tables 1 and 2, among which three threading tools are chosen for the table. Again, the benchmark is conducted with the 20 sequences and all cores in a VM are being utilized by SAGA-Pilot. Obviously, an optimal execution with respect to cost-to-solution is very different from the one with time-to-solution. Also, the results suggest that an optimal solution is not easily achieved unless the parallelization is fully managed. For example, SAGA-Pilot, as default, takes multiple subtasks as a single pilot job and executes them by using all cores available at the moment. Therefore, it is hi1.4xlarge that wastes a lot of computing resources, that is, cores in the second round for 20 subtasks. Nonetheless, the results obtained and shown in Table 6 suggest that the optimization can be pursued by considering the main factor, cost, or computing time, independently. Here, we also note that the real cost could be different from the estimation in Table 6 due to the fact that the pricing scheme is changing over the time and that there is a promotional pricing with free tier. In addition, Amazon pricing, which is per instance-hour for each instance and thus does not allow partial hour usage, could result in slightly more costs. Finally, RHEL pricing is a little bit higher than other open source linux OSs and other costs including the use of EBS volume could be added.

In Table 7, we compare experimental results using SAGA-Pilot and estimated ideal limits using the same 20 sequences out of 110 benchmarks (see Figure 3 for the results with 110 sequences with pfTools). The ideal limit is the time when the benchmark time-to-solution of 20 sequences is divided by the number of cores. The difference shows how the simple parallel implementation using the default parallelism support with SAGA-Pilot works. As expected, our pilot-based results take more time than ideal limits, implying simply the mix of unattainable conditions with the finite number of subtasks and the need of improving the current parallel implementation for further speed-up, in particular, with the instances having multiple cores.

**Figure 6:**
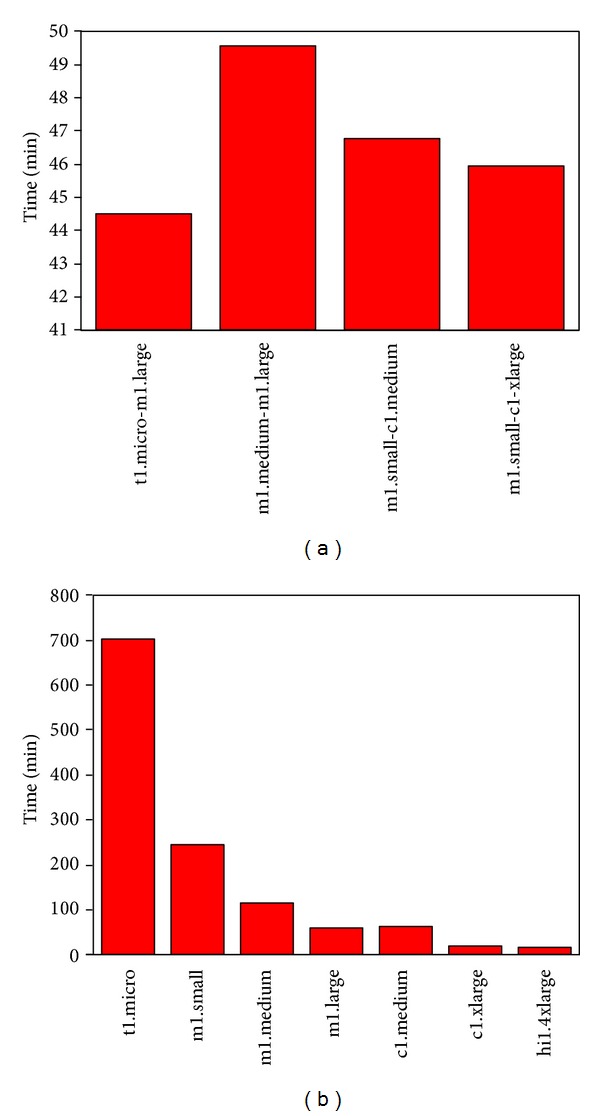
Time-to-solution of each elementary step in the pipeline using 2 heterogeneous VMs (a). Single VM results are presented for comparison (b). Results are obtained with 20 sequences and pfTools are used.

The most significant factor for such discrepancy is understandable with the current implementation for concurrent executions of subtasks. With the efficient job monitoring capacity provided by SAGA-Pilot, all available cores in multicore instances are guaranteed to be utilized and thus contribute speed-up, but there still exists an inefficient utilization of resources. For example, when subtasks corresponding to 20 sequences are distributed into 16 cores, it is highly likely to have idling cores that complete assigned tasks early but need to wait until the last task to be ended by other cores. This is apparently indicated with the fact that the difference from the ideal limit is less significant with the single core instance, m1.small, compared to the dual core c1.large and more apparently to 16-core hi1.4xlarge. This suggests strongly that a better strategy is needed to fully utilize all cores during the time-to-solution. Less computationally demanding tasks with certain tools are more likely affected by nonmajor tasks such as VM launch and another overhead, but overall the expected portion is minimal, suggesting that, to optimize the entire eThread, the key strategy should be the combination of efficient input data parallelization as well as speed-up of tools such as THREADER and “high” computation tools. As we demonstrated, if the case is with mixed instances (see Figure 6), more complicated underlying mechanisms should be considered arising from different CPU performance, the number of cores, and others such as memory. Finally, many features associated with EC2 are not easy to understand with respect to the performance. For example, we observed that the performance of t1.micro is difficult to predict, which can be glimpsed with the two different experiments presented in Figure 5 and Table 4. Two data sets clearly show that t1.micro produces very different outcomes from other instances, in particular, indicated with the relative ratio between chain and domain. Also, in many cases, t1.micro produced unpredictable performance and we suspect, and the information from the explanation from Amazon website, that this is due to a special configuration for this instance to be optimized for low throughput and to be cheaper but not appropriate for computation requiring consistent performance.

### 4.2. Toward Efficient Dynamic Scheduling-Based eThread Pipeline for Genome-Scale Structural Genomics Analysis

Ideally, the best strategy is to implement dynamic scheduling, illustrated in Algorithm 3, that exploits task-level parallelism and data parallelization effectively by dynamically identifying the best resource mapping for upcoming tasks and data transfer. When such an algorithm for dynamic resource mapping exists, SAGA-Pilot can implement it in a straightforward fashion into the pipeline.

Here, to give some insights into such an idea, we describe our exemplary dynamic scheduling, which is currently being evaluated and will be reported as a part of our service elsewhere (see the high-level concept in Algorithm 4). First, by using the obtained 110 sequences benchmark results against each instance type, we train the model for time-to-solution and memory requirement of all threading tools and subtasks relevant for EC2 instance types. This trained model is being used for estimating prospective running times of input sequences. After sorting all input sequences based on their prospective time-to-solution as well as the optimized solution of scheduling all tasks, we start to run them from the longest one in a sorted order. Whenever a subtask is finished, we compare the difference between the real one and the predicted one. If the difference is large enough to leading to the change in an entire time-to-solution, we rearrange the order of remaining tasks to achieve a lower time-to-solution. Therefore, an optimized execution of the pipeline could be achieved by dynamically seeking the best resource mapping.

### 4.3. Future Directions

In addition to the implementation of dynamics scheduling, to further achieve more optimized executions of eThread on EC2 or similar DCI, we could consider other task-level parallelization and data parallelization ideas that we do not present in this work. For example, since BLAST has been developed as multicore or multinode (i.e., MPI support) implementations and data parallelization with BLAST to distribute chain and domain library searches are possible, we can further divide into many subtasks from each elementary task. This gives also a benefit for memory footprint and thus is beneficial to use less power but more number of instances. For example, SAMT2K requires 6 GB RAM due to its BLAST task and could be implemented with such parallel BLAST. Our approach is mostly scale-out at this point but needs to consider scale-up approaches with advanced accelerator techniques such as GPGPU, Intel Phi, and other emerging technologies including IBM Coherence Attach Processor Interface (CAPI).

## 5. Conclusion

eThread is expected to play an important role for genome-scale structural bioinformatics analysis. In spite of its better performance and structural information, in particular, for annotation purposes, required computational demands hinder its usage. To address such a challenge, we developed the SAGA-Pilot-based eThread pipeline and conducted benchmarks aiming at the efficient use of Amazon EC2 infrastructure. With demonstrative examples, we show the support of various data and task-level parallelization scenarios on heterogeneous resources available from Amazon EC2, implying that further optimization ideas including dynamic scheduling could lead to eThread as a practically powerful tool. Other IaaS cloud environments, employing open standards such as OpenStack, are immediately ready to run the eThread pipeline.

Among many potential uses, the eThread pipeline has been developed as a genome-scale annotation tool as a part of an integrative Next-Generation Sequencing (NGS) data analytics. Our continuing effort to build NGS data analytics science gateway, which focuses on utilization of scalable distributed computing and storage resources, is underway (see http://dare.cct.lsu.edu/), which provides a service of the eThread pipeline [33]. Based on our benchmark results and demonstrative experiments, our eThread pipeline is expected to be a viable solution for genome-scale structural bioinformatics and structure-based annotation, particularly, amenable for small genomes such as prokaryotes, even at this moment in a whole-genome scale or personalized medicine for predicting the consequence of mutations occurring in individuals, and easily extensible to utilize other types of distributed cyberinfrastructure (DCI).

## Figures and Tables

**Algorithm 1:**
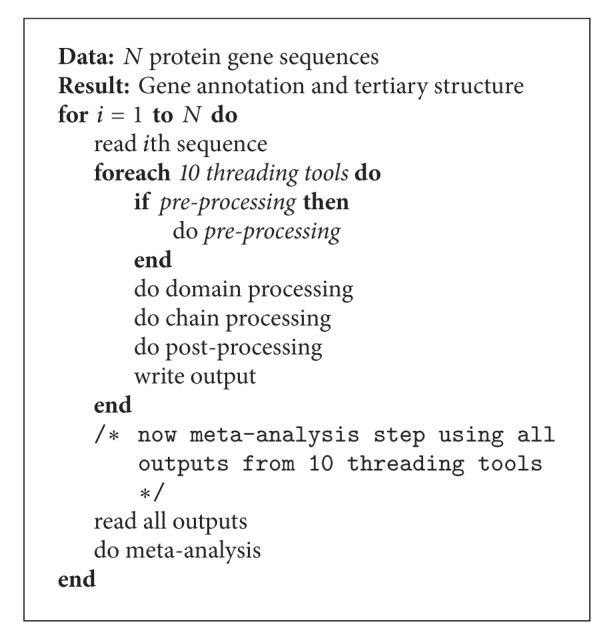
Serial algorithm for eThread.

**Algorithm 2:**
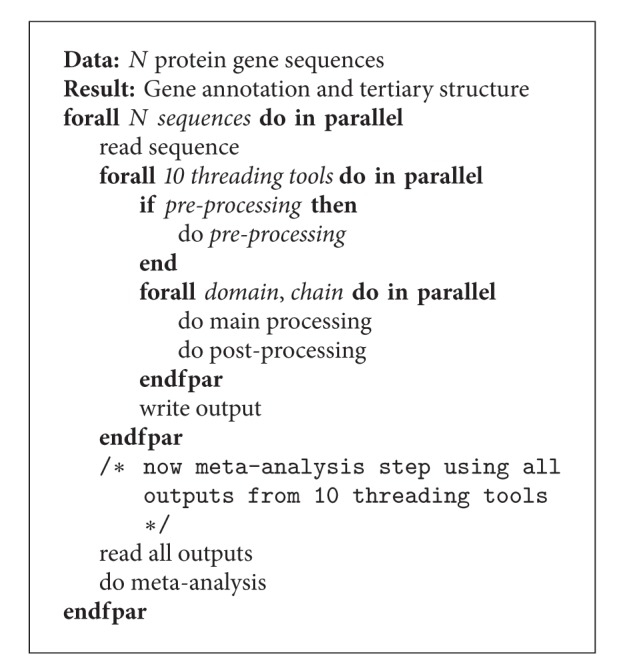
Task-level parallel algorithm for eThread.

**Algorithm 3:**
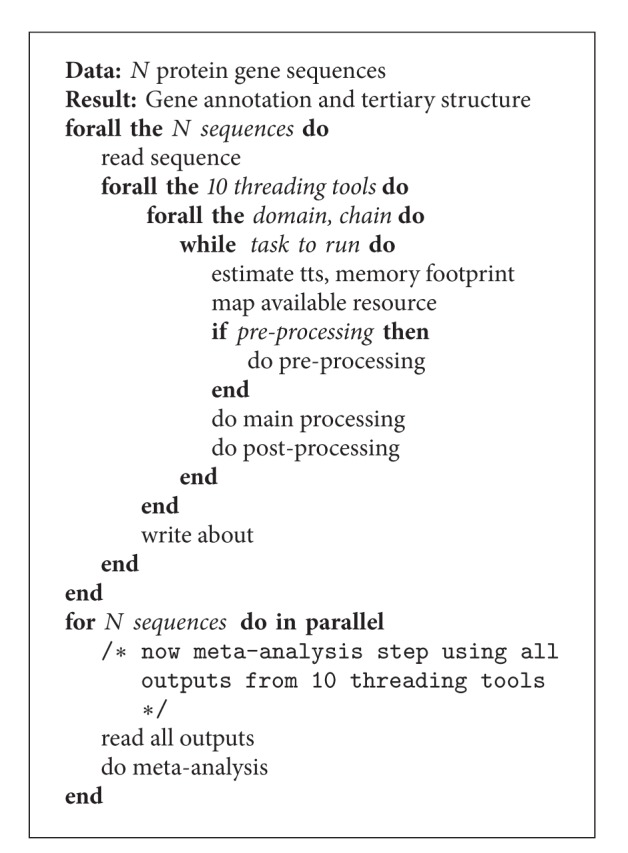
Proposed algorithm combining task-level parallelism and dynamic scheduling for eThread on EC2.

**Algorithm 4:**
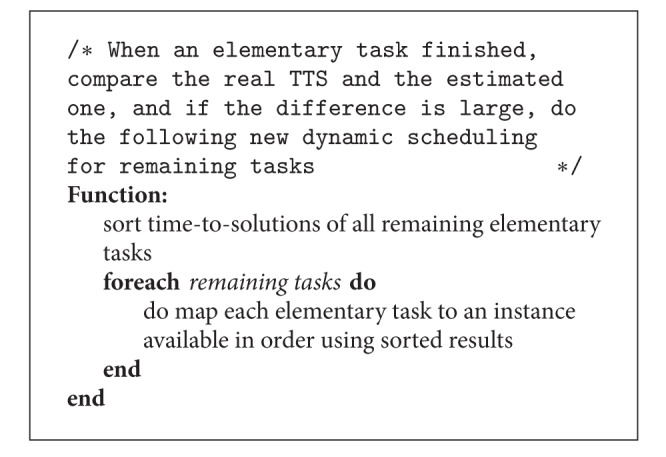
Simple dynamic scheduling implementation for eThread on EC2.

**Table 1:** Threading tools incorporated in eThread and their workflow structures. For the categorization of computational loads and memory requirement, see the text.

Program name (version)	Number of subtasks	Prerequisite	Computational load	Memory requirement
THREADER (3.5)	4	PSIPRED (3.2.1), BLAST (2.2.5)	Highest	Low
SAM-T2K (3.5)	9	BLAST	High	High
HHpred (2.0)	7	BLAST	High	Medium
CS/CSI-BLAST (2.1.0)	4		Low	Low
COMPASS (3.1)	7	BLAST	High	High
pfTools (2.3.4)	4		Medium	Low
pGenTHREADER (8.9)	4	BLAST	High	Low
HMMER (3.1.b1)	4		Low	Low
SPARKS (20050315)	4	BLAST	High	Medium
SP3 (20050315)	4	BLAST	High	Medium

**Table 2:** The summary of EC2 instance types used for this study. For the instance type, E stands for economical, G for general purpose, M for memory-optimized, C for compute-optimized, and S for storage-optimized, following the description from Amazon. Nonsupporting threading tools are identified based on the profiling results of the previous work [28]. The cost information is obtained from the AWS site as of this writing and the unit is $0.02 which is the pricing for t1.micro.

Instance	Type	Number of cores	Memory (GB)	Nonsupport threading tools	Relative cost
t1.micro	E	1	0.613	HHpred, COMPASS, SAM-T2K, pGenThreader, SPARKS, SP3	1
m1.small	G	1	1.7	COMPASS, SAM-T2K, pGenThreader	3
m1.medium	G	1	3.7	SAM-T2K	6
m1.large	M	2	7.5	None	12
c1.medium	C	2	1.7	COMPASS, SAM-T2K, pGenThreader	7.25
c1.xlarge	C	8	7	None	29
hi1.4xlarge	S	16	60.5	None	155

**Table 3:** Benchmark data sets.

Length range (aa)	110 sequences	20 sequences
51–100	10	2
101–150	10	2
151–200	10	2
201–250	10	2
251–300	10	2
301–350	10	2
351–400	10	2
401–450	10	2
451–500	10	1
501–550	10	1
551–600	10	2

**Table 4:** Breaking the time-to-solutions of the main processing step into subtasks. Four subtasks corresponding chain and domain libraries and their postprocessing are measured along with VM launch times. Results are with pfTools. Units are in minutes.

VM launch	Chain	Domain	Chain postprocessing	Domain postprocessing
t1.micro				
1.9	316.5	331.6	33.4	21.5

m1.small				
1.3	137.1	90.1	9.7	7.9

m1.medium				
1.3	62.2	42.9	6.1	4.4

m1.large				
1.2	31.1	21.5	3.4	2.7

c1.medium				
1.3	32.8	22.5	3.9	3.1

c1.xlarge				
1.3	9.5	6.5	1.1	1.2

hi1.4xlarge				
1.5	7.7	5.3	1.3	1.2

**Table 5:** Time for launching an instance. Averaged values of 6 repeated experiments are shown with standard deviation.

Instance	Launching time (min) (standard deviation)
t1.micro	1.99 (0.2)
m1.small	1.86 (0.08)
m1.medium	1.80 (0.15)
m1.large	1.70 (0.08)
c1.medium	1.68 (0.17)
c1.xlarge	1.69 (0.08)
hi1.4xlarge	2.01 (0.16)

**Table 6:** Summary of benchmark results for time-to-solution (TTS) and cost-to-solution (CTS). The 20-sequence data set is used. Among the complete benchmark experimental results obtained for all threading tools, we chose three threading tools here for the sake of space. TTS is in minutes and CTS is in US dollars based on the pricing as of this writing.

VM type	TTS	CTS	TTS	CTS	TTS	CTS
	HMMER		SP3		THREADER	
t1.micro	33.1	0.01	N/A	N/A	96905.8	32.30
m1.small	29.0	0.03	1312.3	1.31	27842.2	27.84
m1.medium	19.6	0.04	670.7	1.34	11551.2	23.10
m1.large	9.8	0.04	458.0	1.83	2897.2	11.59
c1.medium	10.6	0.03	356.7	0.86	6833.8	16.52
c1.xlarge	6.1	0.06	118.6	1.15	2019.3	19.52
hi1.4xlarge	5.8	0.30	105.7	5.46	1552.2	80.20

**Table 7:** Comparison of pipeline-based time-to-solutions with ideal limits. Ideal limits are obtained from the benchmark results of 20 sequences divided by the number of cores in an instance. Units are minutes.

Tools	Pipeline	Ideal limit	Pipeline	Ideal limit	Pipeline	Ideal limit
	m1.small		c1.xlarge		hi1.4xlarge	
SAMT2K	1271.0	1055.7	224.5	65.6	168.3	35.7
SP3	1312.2	1124.4	118.6	68.1	105.7	33.0
CSBLAST	25.2	15.4	6.0	1.23	4.4	0.47
HMMER	29.0	16.0	6.1	1.0	5.8	0.6
pfTools	244.8	226.0	18.3	12.8	15.5	9.2
THREADER	27842.2	23744.0	2019.3	1488.0	1552.2	1090.4
SPARKS	1021.8	1037.7	80.0	54.3	73.3	41.8
